# Trajectories of parent criticism across treatment for youth self‐harm

**DOI:** 10.1111/jcpp.14144

**Published:** 2025-03-24

**Authors:** Madison Aitken, Florence Perquier, Bomi Park, Daniela Carvalho, Alexandra Wright‐Hughes, David Cottrell, Peter Szatmari

**Affiliations:** ^1^ Cundill Centre for Child and Youth Depression Centre for Addiction and Mental Health Toronto ON Canada; ^2^ Department of Psychology York University Toronto ON Canada; ^3^ Department of Psychiatry University of Toronto Toronto ON Canada; ^4^ Schulich School of Medicine & Dentistry Western University London ON Canada; ^5^ School of Medicine University of Leeds Leeds UK; ^6^ Hospital for Sick Children Toronto ON Canada

**Keywords:** Parent–child relationships, adolescence, self‐harm, psychotherapy, expressed emotion

## Abstract

**Background:**

Criticism from parents is a risk factor for poor youth mental health, including self‐harm and limited response to psychosocial interventions. We identified trajectories of change in parent criticism across treatment for youth self‐harm (suicide attempts and non‐suicidal self‐injury) and compared these trajectories on treatment outcomes.

**Methods:**

This is a preregistered secondary analysis of data from the Self‐harm Intervention: Family Therapy trial. Participants (*N* = 831, 11–17 years; 89% girls, 11% boys; 84% White) were clinically referred for self‐harm and randomly assigned to family therapy or usual care. A growth mixture model identified trajectories of parent self‐reported criticism across baseline, 3, and 6 months. Trajectories were compared on youth self‐harm, suicidal ideation, depression, and hopelessness, and parent mental distress (baseline, and change from baseline to 12 and 12–18 months).

**Results:**

Four trajectories of parent criticism were identified: High and remaining elevated despite a small decrease (51.6%); sharply decreasing (7.6%); low/stable (37.2%); and increasing (3.6%). Youth with parents in the high with small decrease class had more severe baseline suicidal behavior. Treatment type was not related to criticism trajectory. Parent mental distress increased in the increasing criticism class. Youth with parents in the increasing class showed less improvement in suicidal ideation at 12‐month follow‐up compared to the high with small decrease and sharply decreasing classes.

**Conclusions:**

Current treatments for youth self‐harm may not reduce parent criticism to subclinical levels. Increasing parent criticism may forecast poorer response to a range of treatments for youth self‐harm and be indicative of increases in parent mental distress.

## Introduction

Self‐harm is prevalent among youth, with approximately 10% of young people in community samples endorsing a history of self‐harm (Hawton, Saunders, & O'Connor, 2012). Rates of self‐harm among youth have increased in the last decade (Curtin & Heron, 2019), and hospital emergency department attendance for self‐harm increased during the COVID‐19 pandemic (Madigan et al., 2023). In the present study, self‐harm refers to intentional self‐injury, regardless of motivation or the presence of suicidal intent (De Leo et al., 2021). Using the broad term of self‐harm reflects the complex relationship between intent and self‐harm, as well as the potential for intent to fluctuate across self‐harm episodes (Holliday, Brennan, & Cottrell, 2020; Witt, Stewart, & Hawton, 2024).

Intervention for youth self‐harm remains a clinical challenge, with relatively few intervention trials, trials of moderate or low quality, and limited effects on repetition of self‐harm in existing studies (Cottrell et al., 2018; Hawton et al., 2015; Witt et al., 2021, 2024). Family conflict is a modifiable risk factor and precipitant for self‐harm among adolescents (Brent et al., 2009; Wilkinson, Kelvin, Roberts, Dubicka, & Goodyer, 2011), leading to increasing interest in the effectiveness of family interventions for youth self‐harm in recent years (Asarnow, Hughes, Babeva, & Sugar, 2017; Diamond et al., 2019; Korczak et al., 2020).

One aspect of the family environment consistently related to youth self‐harm is how critical parents are toward the youth (Wedig & Nock, 2007). Criticism is a key dimension of expressed emotion, an indicator of family relationship functioning that is associated with the severity and relapse of mental illness across the spectrum of psychopathology (Hooley, 2007; Wedig & Nock, 2007). Criticism suggests the presence of problems in the parent–youth relationship and has a bidirectional association with youth mental health (Hooley, 2007). Criticism is a prognostic indicator of future psychopathology across childhood and adolescence (Peris & Miklowitz, 2015; Silk et al., 2009); however, adolescent psychopathology also predicts increases in parent criticism over time (Nelemans, Hale, Branje, Hawk, & Meeus, 2014), which may reflect increasing frustration due to failed efforts to help the young person function better (Hooley, 2007). Criticism may therefore also be an indicator of family members' response to emerging psychopathology in a young person (Peris & Miklowitz, 2015). Importantly, criticism is not considered a stable trait and shows only moderate stability across time (Frye & Garber, 2005).

High levels of parent criticism have been associated with more self‐harm and suicidal ideation in community and clinical samples of youth (Ellis et al., 2014; Wedig & Nock, 2007). While most studies examining parent criticism and youth self‐harm are cross‐sectional, recent evidence from a pre‐adolescent sample using intensive longitudinal data suggests that increases in parent criticism predict a subsequent increase in suicidal thoughts and behaviors (Thompson et al., 2024). Parent criticism may contribute to youth self‐harm by increasing youth self‐criticism (Baetens et al., 2015), emotion dysregulation (Berla et al., 2022), or depressive symptoms (Peris & Miklowitz, 2015), which may in turn lead to greater criticism (Frye & Garber, 2005). High levels of parent criticism also predict a poorer response to psychosocial interventions for youth depression and other disorders (Peris & Miklowitz, 2015); therefore, parent criticism may be an important target for psychosocial interventions for youth, including those who self‐harm.

### Family interventions for youth self‐harm

Family interventions may decrease youth self‐harm by strengthening protective factors, such as parents' ability to respond to suicide risk, ensure youth safety, and provide effective supervision and emotional support (Asarnow et al., 2017; Diamond et al., 2010). Family interventions may also reduce family conflict (Fortune, Cottrell, & Fife, 2016), an important risk factor for youth self‐harm that is associated with poorer response to psychotherapy in youth at risk for self‐harm (Brent et al., 2009; Diamond et al., 2010; Wilkinson et al., 2011). A recent review reported that the inclusion of family or caregivers in the intervention was a common feature across effective interventions for youth self‐harm (Meza, Zullo, Vargas, Ougrin, & Asarnow, 2023). Studies of other clinical populations have shown that family therapy decreases criticism among caregivers in systemic family therapy for child emotional and behavioral problems (Vostanis, Burnham, & Harris, 1992), family members of adults with depression in a multifamily psychoeducation intervention (Katsuki et al., 2011), parents and youth in family focused therapy for youth at clinical high risk for psychosis (O'Brien et al., 2014), and parents in family therapy for adolescents with anorexia (Eisler et al., 2000). While family therapy may reduce criticism in family members of individuals with mental health difficulties, there is little information on how parent criticism changes during family therapy, and previous studies have not focused on clinical populations involving youth who self‐harm.

### SHIFT trial

The Self‐Harm Intervention: Family Therapy (SHIFT; Cottrell et al., 2018; Wright‐Hughes et al., 2015) trial was a pragmatic, multicenter randomized controlled trial comparing family therapy with treatment as usual (TAU) for youth self‐harm. Family therapy involved a manualized, systemic approach and approximately eight 75‐min sessions over 6 months (Wright‐Hughes et al., 2015). The family therapy intervention was based on a modified version of an existing manual (Pote, Stratton, Cottrell, Shapiro, & Boston, 2003) that was adapted to ensure an adequate focus in early sessions on self‐harm and risk assessment. It was manualized and designed to be delivered by experienced, qualified, family therapists who were allowed flexibility to deliver what was a complex intervention. The intervention was based on a systemic orientation, which focused on exploring and changing unhelpful patterns of interactions within families, as well as developing more positive narratives (Boston, Eisler, & Cottrell, 2018). Reducing the frequency and severity of self‐harm was a central goal of family therapy (Boston et al., 2018). Relevant to the present study, family therapy prioritized addressing critical, hostile, or invalidating communication (Boston et al., 2018). Examples of approaches used to address maladaptive communication styles include circular questions, or expanding the time frame to consider how patterns emerged over time (Boston et al., 2018). TAU involved routine mental health care provided within the participant's local mental health service. There were no restrictions on TAU, and clinicians employed a range of individual and family‐based approaches (Cottrell et al., 2018). Consistent with evidence that self‐harm is more common among girls than boys (Miranda‐Mendizabal et al., 2019; Valencia‐Agudo, Burcher, Ezpeleta, & Kramer, 2018), the majority of participants in the SHIFT trial were girls.

Family therapy was not associated with significantly greater reductions in hospital attendance for self‐harm at 18‐month follow‐up compared to TAU (Cottrell et al., 2018); however, moderation analyses suggested heterogeneity in treatment outcomes was based on a variety of factors, including family affective involvement (Cottrell et al., 2018). By examining aspects of the family emotional climate in greater detail, including how they may change across treatment, we may be able to understand heterogeneity in treatment outcomes for youth self‐harm.

### Present study

The variable‐oriented analyses used in clinical trials, in which effectiveness is determined through average treatment effects across individuals who received a specific treatment, implicitly assume a similar treatment effect across heterogeneous patient characteristics (Davies et al., 2020). In contrast, person‐oriented analyses can identify discrete patterns within a larger sample, making it possible to detect subgroups by their treatment response and to examine how and for whom various interventions are effective (Lundh & Falkenström, 2019). In the case of parent/caregiver criticism, studies have generally examined group‐level changes across treatment (Eisler et al., 2000; Katsuki et al., 2011; O'Brien, Miklowitz, & Cannon, 2015; Shimazu et al., 2011), which assumes that change is homogeneous; however, at least one study in youth with anorexia suggests that parents show heterogeneous changes in criticism levels across treatment (Allan, Le Grange, Sawyer, McLean, & Hughes, 2018).

The present study uses growth mixture modeling, a person‐oriented analysis, to identify patterns of change in parent criticism across treatment for youth self‐harm in the SHIFT trial (Cottrell et al., 2018). We compare the resulting trajectories of parent criticism on treatment condition (family therapy vs. TAU) and youth and parent mental health at baseline, as well as youth treatment outcomes and parent mental distress at follow‐up. Our preregistered hypotheses for these secondary analyses were: (a) Distinct trajectories of change in parent criticism will be identified; (b) the probability of belonging to the trajectories will differ by treatment type (family therapy vs. TAU); (c) trajectories characterized by higher and more persistent criticism will be associated with more severe youth hopelessness, depression, self‐harm, and suicidal ideation, and parent mental distress, at baseline; (d) decreasing parent criticism will be associated with better youth treatment outcomes and with decreases in parent mental distress at 12 and 18 months.

## Methods

We carried out a preregistered secondary analysis of data from the SHIFT trial (Cottrell et al., 2018; Wright‐Hughes et al., 2015), including parent self‐reported criticism during the treatment phase (baseline, 3, and 6 months), youth self‐reported mental health problems and self‐harm, and parent self‐reported mental health problems, at baseline and follow‐up (12 and 18 months).

### Participants

Participants were recruited from 40 Child and Adolescent Mental Health Services in the United Kingdom. All participants had self‐harmed prior to being assessed and had self‐harm as a key feature of their current presentation (Wright‐Hughes et al., 2015). A total of 832 youth aged 11–17 years (*M*_age_ = 14.3; *SD*_age_ = 1.4; 89% girls, 11% boys; *n* = 415 in family therapy, *n* = 417 in TAU; 84% White, 7% Black, 4% Asian, 5% another ethnicity) and their caregivers (86% mothers, 11% fathers, 1% guardians, 0.6% step‐mothers, 0.2% step‐fathers; 0.2% foster parents) participated (Cottrell et al., 2018). One participant was missing parent criticism ratings at all time points and was excluded from the analysis (*N* = 831).

### Measures

#### Background and demographics

Information was collected at baseline through interviews and case notes, including demographic information, current psychotropic medication use, and history of abuse (Wright‐Hughes et al., 2015).

#### Criticism

Parent ratings on the 10‐item Criticism subscale of the Family Questionnaire (Wiedemann, Rayki, Feinstein, & Hahlweg, 2002) were used, each rated on a 4‐point scale. The Family Questionnaire has demonstrated internal consistency, convergence with established measures of expressed emotion, and sensitivity to the effects of parent/family interventions (McEvoy et al., 2019; Wiedemann et al., 2002). Scores of 24 and above indicate high levels of criticism (Wiedemann et al., 2002). The primary caregiver completed the Family Questionnaire at baseline and at 3 and 6 months (during the intervention phase). Internal consistency was acceptable (αs = .92, .92, and .93 at baseline, 3, and 6 months).

#### Youth mental health

Three measures of youth mental health completed at baseline and 12 and 18 months were used: (a) Hopelessness Scale for Children (Kazdin, Rodgers, & Colbus, 1986), consisting of 17 self‐report yes/no items; (b) revised Children's Depression Rating Scale (Poznanski et al., 1984), a clinician‐rated measure of depressive symptoms and non‐verbal behaviors; and (c) Beck Scale for Suicide Ideation (Beck, Kovacs, & Weissman, 1979), containing 21 self‐report items on the severity of suicidal thoughts rated on a 3‐point scale. All three measures have demonstrated reliability and validity (Beck et al., 1979; Mayes, Bernstein, Haley, Kennard, & Emslie, 2010; Thurber, Hollingsworth, & Miller, 1996). Internal consistency was acceptable for the Hopelessness Scale for Children (α = .88, .90, and .91), revised Children's Depression Rating Scale (α = .81, .87, and .90), and Beck Scale for Suicide Ideation (α = .89, .88, and .89) at baseline, 12, and 18 months.

#### Youth self‐harm

Information on the episode of self‐harm that brought the youth into mental health services was gathered with the Suicide Attempt Self‐Injury Interview (Linehan, Comtois, Brown, Heard, & Wagner, 2006), which has demonstrated reliability and concurrent validity, primarily in adults (Borschmann, Hogg, Phillips, & Moran, 2012; Linehan et al., 2006). Variables used included probability of intervention (low vs. high), intent to die (yes/no), lethality of the self‐harm method (low, moderate, high), and endorsement of emotional relief or interpersonal influence reasons for self‐harm. The presence or absence of self‐harm between baseline and 12 months and 12 and 18 months was also extracted; specifically, youth were asked whether they had ‘deliberately harmed or injured [themselves] or attempted suicide’.

#### Parent mental distress

Parents completed the General Health Questionnaire‐12 (GHQ‐12; Goldberg & Hillier, 1979), a measure of mental distress, with each statement rated on a 4‐point scale over the past 2 weeks, at baseline, 12 months, and 18 months. The GHQ‐12 has demonstrated a unidimensional factor structure, internal consistency, and concurrent validity (Romppel, Braehler, Roth, & Glaesmer, 2013). Internal consistency was good (α = .92, .93, and .93 at baseline, 12, and 18 months).

### Analyses

Our analysis followed a plan preregistered with the Open Science Framework https://osf.io/mnrgc. Deviations are described in the Supporting Information. We follow the Guidelines for Reporting on Latent Trajectory Studies (van de Schoot, Sijbrandij, Winter, Depaoli, & Vermunt, 2017; see Table S1).

Analyses were carried out using Mplus 8.3 (Muthén & Muthén, 2017). We conducted a growth mixture model analysis of parent criticism with variances for intercepts and slopes fixed across classes, 1,000 random starting values, 50 final stage optimizations, and no covariates (hypothesis 1). Time points were equally spaced and corresponded to baseline, 3‐ and 6‐months post‐randomization. Parameters were fixed as needed to obtain a model that converged (see Supporting Information). The best‐fitting model was selected based on the Bayesian information criterion, bootstrap likelihood ratio test, and the size and meaningfulness of the classes (Muthén & Muthén, 2000). Alternative specifications, including a latent growth curve analysis and a nonlinear longitudinal latent class analysis, were examined (see Supporting Information).

Once the best‐fitting number of classes was identified, we compared the classes (dependent variable) on treatment condition (independent variable: family therapy vs. TAU) using multinomial logistic regression with the three‐step method (R3STEP; hypothesis 2; Asparouhov & Muthén, 2014). We similarly compared the classes on baseline variables (severity of youth suicidal ideation, depression, and hopelessness; characteristics of youth self‐harm; and parent mental health), controlling for youth age, sex, and history of abuse due to their potential association with differences in youth mental health and self‐harm (hypothesis 3). Lastly, to test hypothesis 4, we compared the classes (independent variable) on youth treatment outcomes and on parent mental health outcomes (dependent variables; change from baseline to 12‐month follow‐up, from 12‐ to 18‐month follow‐up, and from baseline to 18‐month follow‐up) using the three‐step method (DE3STEP for continuous variables, BCH method for binary variables; covariates cannot be included in these models; Asparouhov & Muthen, 2021).

We examined patterns of missing data and handled missing data with a pattern mixture model to account for data not missing at random (Muthén, Asparouhov, Hunter, & Leuchter, 2011), full information maximum likelihood estimation (hypotheses 1, 2, and 4), or multiple imputation (hypothesis 3; see Supporting Information for details and rationale). A *p*‐value < .05 on two‐tailed tests was considered significant. Comparison of classes on treatment condition and baseline variables (hypotheses 2 & 3) used the low/stable class as a reference class; therefore, no correction was made for multiple pairwise comparisons. Comparisons of classes on treatment outcomes used a corrected α = .017 (.05/3, following our preregistered plan to compare change across three time intervals in a pairwise manner).

## Results

### Preliminary analyses

Rates of missing data for parent criticism were *n* = 1 at baseline; *n* = 393 (47%) at 3 months; and *n* = 472 at 6 months (57%). Rates of missing data on outcome measures were *n*s = 430–449 (46–48%) at 12 months and *n*s = 302–321 (61–64%) at 18 months. Data were considered missing not at random because participants missing parent criticism ratings at 3 and 6 months had significantly higher baseline parent criticism than participants with criticism ratings available at 3 and/or 6 months; *t*(829) = 2.45, *p* = .014 at 3 months; *t*(829) = 3.21, *p* = .001 at 6 months. In addition, participants with missing criticism data at 3 months or 6 months were significantly more likely to be in TAU than family therapy, χ^2^(1) = 9.36, *p* = .002, and χ^2^(1) = 18.15, *p* < .001 for those missing at 3 and 6 months, respectively. Participants with missing criticism ratings had significantly higher baseline parent mental distress and endorsed less emotion relief functions of self‐harm and greater likelihood of having communicated their suicide intent to someone. Participants missing one or more outcome variables at 12 months were more likely to be in TAU than family therapy, were older, and had parents with higher baseline parent mental distress than participants with complete data at 12 months. Participants missing one or more outcome variables at 18 months were more likely to be in TAU, to be girls, to have experienced physical abuse, and had higher baseline parent criticism scores than participants with complete data at 18 months. There were no other significant differences between those with missing and complete data at 12 and 18 months (see Supporting Information).

Variability in the timing of measurements around the nominal time points in the growth mixture model was relatively small (baseline *M* = −0.03, *SD* = 0.05; 3 months *M* = 3.37, *SD* = 0.41; 6 months *M* = 6.37, *SD* = 0.49). Criticism scores were approximately normally distributed at all time points. Descriptive statistics are presented in Table 1.

**Table 1:** Descriptive statistics for sample and study variables

Variable	*M* (SD) or %				
	Baseline^a^	3 months^b^	6 months^c^	12 months^d^	18 months^e^
Age (years)	14.3 (1.4)				
Gender					
Girls	89				
Boys	11				
Race/Ethnicity					
White	84				
Black	7				
Asian	4				
Another ethnicity	5				
Psychiatric medication	5.0	–	–	–	–
History of abuse					
Physical by parent	24.0	–	–	–	–
Physical leaving marks	22.7	–	–	–	–
Sexual	16.7	–	–	–	–
Parent criticism	25.72 (7.01)	23.85 (6.55)	22.76 (6.66)	–	–
Depression	48.69 (13.74)	–	–	36.82 (13.78)	34.38 (14.57)
Suicidal ideation	10.60 (9.17)	–	–	5.11 (7.55)	4.86 (7.75)
Hopelessness	7.51 (4.25)	–	–	5.04 (4.13)	4.71 (4.13)
Parent mental distress	18.16 (7.16)	–	–	13.22 (6.62)	13.27 (6.60)
Deliberate self‐harm	100.0	–	–	68.8	41.5
Characteristics of index self‐injury episode					
For emotional relief	3.37 (1.78)	–	–	–	–
For interpersonal influence	0.96 (1.58)	–	–	–	–
Intent to die	49.5	–	–	–	–
Communicated intent	27.5				
Lethality					
Low	75.5	–	–	–	–
Moderate	21.8	–	–	–	–
High	2.7	–	–	–	–
Low intervention probability	20.7	–	–	–	–

^a^ *n* = 831.

^b^ *n* = 439.

^c^ *n* = 360.

^d^ *n*s = 431–459.

^e^ *n*s = 369–392.

### Growth mixture model

**Figure 1:**
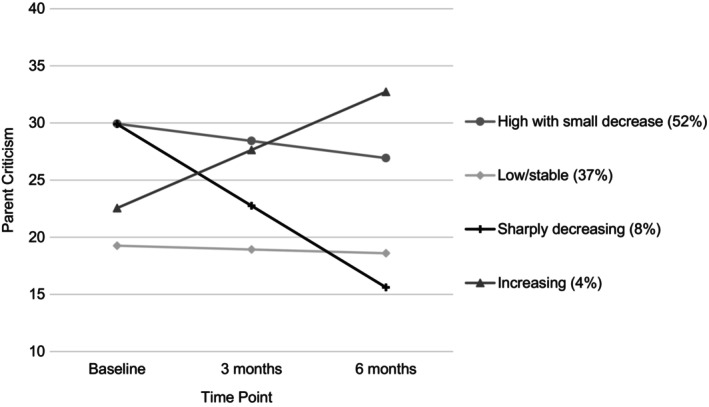
Growth mixture model of parent criticism across treatment (*N* = 831)

Fit statistics for all models are presented in Table 2. Figure S1 shows estimated trajectories for each model. A 4‐class model fit the data best (see Figure 1 and Table S4) based on fit statistics. In addition, the fourth class was distinct from the classes in the three‐class model, suggesting it identifies a unique and potentially clinically relevant group of parents. The four‐class model consisted of the following classes: High with small decrease (51.6% of sample; average posterior probability = 0.80), which showed a small but significant decrease in criticism but remained in the elevated range (slope *M* = −1.50, *SE* = 0.25, *p* < .001); sharply decreasing (7.6% of sample; average posterior probability = 0.79), which showed a large, significant decrease in criticism (slope *M* = −7.14, *SE* = 0.84, *p* < .001); low/stable (37.2% of sample; average posterior probability = 0.89), which had low criticism at baseline and did not change significantly (slope *M* = −0.33, *SE* = 0.23, *p* = .15); and increasing (3.6% of sample; average posterior probability = 0.76), which showed a large, significant increase in criticism (slope *M* = 5.09, *SE* = 0.87, *p* < .001). See Figure S2 for observed individual trajectories by class for the four‐class model. To account for the relatively large amounts of missing data and data not being missing at random, we conducted a sensitivity analysis using a pattern mixture model. The pattern mixture model resulted in a similar four‐class model (see Supporting Information); therefore, potential bias due to missing data appears to be minimal.

**Table 2:** Growth mixture model fit statistics (*n* = 831)

Classes	BIC	BLRT *p*	Entropy	Class size
1	10,371.71	–	–	–
2	10,347.62	<.001	0.591	c_1_ = 61.6% c_2_ = 38.4%
3	10,353.09	<.001	0.597	c_1_ = 54.3% c_2_ = 39.3% c_3_ = 6.4%
**4**	**10,348.35**	**<.001**	**0.631**	**c**_**1**_ **= 51.6%** **c**_**2**_ **= 7.6%** **c**_**3**_ **= 37.2%** **c**_**4**_ **= 3.6%**
5	10,360.58	.109	0.518	c_1_ = 34.8% c_2_ = 31.8% c_3_ = 3.4% c_4_ = 5.6% c_5_ = 24.4%

Bolded row indicates the best‐fitting model based on BIC, BLRT, class size, and theoretical considerations. BIC, Bayesian information criterion; BLRT, bootstrap likelihood ratio test.

### Comparing classes on baseline characteristics

Comparisons of parent criticism classes on baseline variables are presented in Table 3. There were no significant associations between class membership and treatment assignment (family therapy vs. TAU). There were no significant differences across classes in the severity of youth depression, suicidal ideation, or hopelessness. In terms of characteristics of the index self‐harm incident, youth with parents in the high with small decrease criticism class endorsed significantly less emotional relief functions of self‐harm, greater intent to die, and the use of more lethal means compared to youth whose parents were in the low/stable criticism class. In addition, youth with parents in the increasing criticism class endorsed significantly more lethal means of self‐harm compared to youth with parents in the low/stable criticism class. Classes did not differ significantly in youth endorsement of interpersonal influence reasons for self‐harming or the probability of their self‐harm attempt being intervened upon. Parents in the high with small decrease criticism class endorsed significantly greater mental distress themselves at baseline compared to parents in the low/stable criticism class. Associations between control variables and class membership are reported in the Supporting Information (Table S5).

**Table 3:** Multinomial logistic regressions of criticism class membership on baseline variables (*n* = 831)

Baseline variable	High with small decrease			Sharply decreasing			Increasing		
	Estimate	*SE*	*p*	Estimate	*SE*	*p*	Estimate	*SE*	*p*
Treatment as usual	−0.232	0.214	.278	−0.465	0.534	.384	−0.366	0.731	.616
Depression^a^	−0.008	0.010	.413	0.017	0.034	.622	−0.041	0.030	.175
Suicidal ideation^a^	0.015	0.015	.308	−0.045	0.056	.421	0.020	0.045	.655
Hopelessness^a^	0.015	0.031	.635	−0.106	0.095	.262	−0.036	0.084	.666
Parent mental distress^b^	**0.151**	**0.025**	**<.001**	0.072	0.070	.303	0.052	0.078	.507
Characteristics of index self‐injury episode									
For emotional relief^a^	**−0.191**	**0.080**	.**017**	0.140	0.243	.563	−0.306	0.326	.349
For interpersonal influence^b^	0.139	0.084	.097	0.288	0.153	.060	−0.096	0.907	.916
Intent to die^a^	**0.496**	**0.237**	.**036**	0.330	0.562	.557	0.877	0.827	.289
Lethality^a^	**0.592**	**0.256**	.**021**	−0.089	0.896	.921	**1.365**	**0.592**	.**021**
Low intervention probability^a^	−0.299	0.298	.314	−0.119	0.682	.861	−0.157	1.193	.895

Bold values indicate a significant difference. Reference class is the low/stable class. Variables were entered in separate analyses along with control variables.

^a^ Analysis controlled for youth age, gender, and abuse history.

^b^ Analysis controlled for youth age and abuse history. Gender omitted due to issues of convergence/small cells.

### Treatment response differences across classes

Youth with parents in the high with small decrease (χ^2^ = 21.74, *p* < .001) and sharply decreasing (χ^2^ = 5.83, *p* = .016) criticism classes showed significantly larger decreases in suicidal ideation from baseline to 12‐month follow‐up compared to youth with parents in the increasing criticism class. Parents in the increasing criticism class showed an increase in mental distress themselves from baseline to 12 months and differed significantly from parents in the high with small decrease (χ^2^ = 11.33, *p* = .001) and low/stable (χ^2^ = 5.94, *p* = .015) classes, who showed decreases in their own mental distress. There were no other significant differences between classes, including change in youth depression, hopelessness, or the presence or absence of self‐harm incidents from baseline to 12 months (see Table 4).

**Table 4:** Comparison of classes on treatment outcomes using the three‐step method for distal outcomes (*n* = 831)

Class	Dependent variable			
	Depression	Suicidal ideation	Parent mental distress	Presence of self‐harm
*Baseline to 12 months*	1.33 *p* = .72	**27.56** ***p* < .001**	**17.03** ***p* = .001**	0.90 *p* = .83
High with small decrease	−10.11 (1.96)	−6.44^a^ (0.77)	−5.29^a^ (1.04)	–
Sharply decreasing	−22.48 (10.77)	‐ 6.63^b^ (2.12)	−4.88 (5.46)	–
Increasing	−5.98 (11.59)	−0.92^a,b^ (1.11)	1.22^a,b^ (1.57)	–
Low/stable	−11.16 (1.55)	−4.27 (0.95)	−2.79^b^ (0.55)	–
*12–18 months*	–	6.33 *p* = .10	1.72 *p* = .63	1.40 *p* = .71
High with small decrease	–	0.33 (0.61)	−0.31 (0.84)	–
Sharply decreasing	–	−2.20 (1.66)	0.11 (4.06)	–
Increasing	–	1.08 (1.93)	0.12 (6.05)	–
Low/stable	–	−1.93 (0.82)	0.97 (0.64)	–
*Baseline to 18 months*	0.76 *p* = .859	**15.51** ***p* = .001**	**19.32** ***p* < .001**	–
High with small decrease	−12.87 (1.80)	−5.74^a^ (1.00)	−6.11^a,b^ (0.93)	–
Sharply decreasing	−19.02 (8.27)	−13.09^b,c^ (2.84)	−7.13 (3.65)	–
Increasing	−1.96 (21.01)	−0.29^a,c,d^ (1.86)	1.66^b^ (2.49)	–
Low/stable	−14.30 (2.12)	−5.43^b,d^ (0.95)	−2.41^a^ (0.68)	–

Superscript letters within the same column and time period indicate significant differences between classes. Means and standard errors for the presence of self‐harm are not reported because they cannot be interpreted in the standard way due to the use of the BCH method for the three‐step analysis of this variable. The significance value applied was *p* < .017.

When we examined class differences in change between 12‐ and 18‐month follow‐up, there were no significant differences between classes in change in youth suicidal ideation, hopelessness, the presence or absence of self‐harm incidents, or in parent mental distress (see Table 4). Although we also compared change in depression from 12 to 18 months across the classes, estimates were not trustworthy due to high classification error once the distal outcome variable was included and therefore are not interpreted.

Finally, when we examined change from baseline to 18‐month follow‐up, classes differed significantly on change in suicidal ideation and change in parent mental distress. Specifically, the increasing criticism class showed a smaller decrease in suicidal ideation compared to the high with small decrease (χ^2^ = 6.16, *p* = .013), sharply decreasing (χ^2^ = 14.20, *p* < .001), and low/stable (χ^2^ = 5.92, *p* = .015) classes. In addition, the sharply decreasing class showed a larger decrease in suicidal ideation than the low/stable class (χ^2^ = 6.55, *p* = .010). For parent mental distress, parents in the increasing criticism class showed an increase in mental distress from baseline to 18 months and differed significantly from parents in the high with small decrease class (χ^2^ = 8.09, *p* = .004). In addition, the high with small decrease class showed a larger decrease than the low/stable class (χ^2^ = 8.53, *p* = .004). There were no other significant differences between classes, including for change in youth depression (see Table 4).

## Discussion

Criticism by parents is an important aspect of expressed emotion within the family and a predictor of self‐harm and of youth treatment outcomes for eating disorders, obsessive‐compulsive disorder, bipolar disorder, and depression (Peris & Miklowitz, 2015). We examined change in parent criticism during treatment for youth self‐harm using data from a large, pragmatic clinical trial (Cottrell et al., 2018). We also tested the extent to which changes in parent criticism were associated with differences in youth self‐harm characteristics and with differences in youth treatment outcomes. Our results highlight the variability in how parent criticism changes across treatment for youth self‐harm. Growth mixture modeling identified small subgroups of parents who showed significant increases or decreases in criticism across treatment; however, the majority of parents showed persistently high criticism across treatment (despite a small decrease). Persistently high parent criticism across treatment was associated with differences in the characteristics of youth self‐harm. Increases in parent criticism also predicted less improvement in youth suicidal ideation in the post‐treatment period. Moreover, parent criticism was closely related to parents' own mental distress.

A large proportion of parents of youth seeking treatment for self‐harm were highly critical of their youth and remained so across treatment. These findings suggest that current treatments for youth self‐harm, even when focused on the family environment, may not reduce parent criticism to adaptive levels in many families. We also found that youth whose parents had persistently high criticism tended to begin treatment with a constellation of characteristics indicative of more severe suicidal behavior, including the use of more lethal means, more endorsement of intent to die, and less endorsement of self‐harming for emotion relief, consistent with evidence that family relationship problems are a risk factor for youth suicide attempts (Wilkinson et al., 2011). It is possible that clinicians working with these families prioritized reducing youth self‐harm frequency and severity and/or addressing suicide risk (DeCou, Comtois, & Landes, 2019), and addressing parent criticism may have been a lower priority. There is some evidence that conjoint family treatment, such as that used in the family therapy arm, leads to smaller decreases in maternal criticism than parent‐focused treatments (Allan et al., 2018). Further research, including clinical trials, is needed to determine whether parent‐focused interventions targeting criticism specifically may be beneficial in the context of youth self‐harm.

Parental criticism was also related to higher levels of parent mental distress. Parents who showed persistently high levels of criticism toward their youth reported more mental distress themselves at baseline. In addition, parents who showed an increase in criticism across treatment showed significantly greater increases in their own mental distress across treatment and follow‐up. Our results are consistent with evidence from previous studies that parents with higher levels of depression express more criticism toward their youth (Gibb, Uhrlass, & Grassia, 2010); however, parents who showed persistently high levels of criticism did show some of the largest decreases in their own mental distress in our study, suggesting that parent mental distress and criticism are separable phenomena. Therefore, once established, a pattern of high parent criticism may persist even after parents' own mental health improves.

While many parents showed a pattern that involved elevated criticism at some point during treatment, approximately one‐third had consistently low levels of criticism toward their youth. Several variable‐oriented studies have demonstrated an association between youth self‐harm and higher levels of parent criticism, which appears to be mediated through adolescent self‐criticism (Baetens et al., 2015; Wedig & Nock, 2007). The present person‐oriented results indicate that, while parent criticism is common when youth self‐harm, not all parents exhibit this profile of expressed emotion; therefore, a more individualized consideration of parent criticism may be informative in research and in clinical case conceptualizations regarding youth self‐harm.

In terms of treatment outcomes, contrary to our hypotheses, we did not find that the probability of belonging to any of the trajectories of parent criticism differed by treatment type (family therapy or TAU). Most previous studies that have focused on parent criticism as an outcome have used family therapy (Eisler et al., 2000; O'Brien et al., 2015) or psychoeducational approaches with families (Katsuki et al., 2011). The present results are novel in showing that family therapy and usual outpatient care were equally likely to result in any of the four patterns of parent criticism across treatment (high with small decrease, low/stable, increasing, or sharply decreasing). Only a small proportion (8%) of parents in the present study showed a large decrease in criticism, moving from high to typical levels by 6 months after the start of treatment. Therefore, further research is needed to determine how best to address high or increasing levels of parent criticism. For example, parent training in communication skills and/or problem‐solving may be necessary to decrease parent criticism (Peris & Miklowitz, 2015).

We also examined differences in youth mental health outcomes from baseline to follow‐up based on the pattern of parent criticism across treatment. Unexpectedly, youth whose parents showed persistently high criticism with a small decrease across treatment did not differ from youth whose parents were low in criticism in terms of change in suicidal ideation, depression symptoms, or hopelessness in the year following the end of treatment. These results contrast with evidence that parent criticism is a risk factor for youth psychopathology persistence and recurrence (Butzlaff & Hooley, 1998). Our unexpected results could be explained in several ways. First, criticism levels of parents in the high class did show a small but significant decrease, which may suggest that even small improvements in parent criticism support improvements in youth mental health. Second, both family therapy and usual care were flexible (Cottrell et al., 2018), allowing therapists to use other approaches to mitigate the effects of high parent criticism on youth mental health. We also used parent self‐report of criticism, whereas most previous studies used coded interviews or speech samples (Butzlaff & Hooley, 1998). Parents in the present study may have become aware of their critical behaviors and therefore may have been sensitized to endorse more criticism, even if their critical behavior decreased.

Finally, the class of youth whose parents' criticism increased across treatment showed less improvement in suicidal ideation across baseline and extended follow‐up at 12 and 18 months than youth whose parents' criticism decreased sharply, was high with a small decrease, or was stable and low across treatment (the latter only showing significant differences in suicidal ideation change from baseline to 18 months). Participating in therapy may change established parent–youth interaction patterns, creating opportunities for therapy effects (Hayes & Andrews, 2020). It is possible that this subgroup of families changed in such a way during therapy that parents developed more critical views. A small but important subset of adults report increases in family conflict following psychotherapy (Ladwig, Rief, & Nestoriuc, 2014), and increased criticism has been observed in approximately a quarter of mothers in conjoint family‐based treatment for youth anorexia (Allan et al., 2018). Families whose criticism increased may also have been managing other stressors that contributed to an increase in parent criticism or may have been responding to a lack of improvement in youth suicidal ideation. The latter two interpretations are consistent with the observed increase in mental distress in parents whose criticism increased across treatment. While these interpretations are speculative, our findings suggest that it may be important to monitor parent criticism and other psychological adverse events across treatment for youth self‐harm (e.g., through measurement‐based care or other methods), as increases in criticism forecast poorer post‐treatment outcomes in terms of youth suicidal ideation.

### Limitations

Several limitations should be considered in interpreting the present findings. First, as this was a pragmatic trial in outpatient clinics, rates of missing data beyond baseline were high. Of note, higher baseline parent criticism was associated with greater likelihood of missing data at subsequent time points, and missing data were more common in TAU than in family therapy. While we took appropriate steps to address missing data, we cannot rule out potential effects of missing data on our results, particularly in the analysis of treatment outcomes; however, there were few differences between those with and without 12‐month outcome data on variables used in the analysis. Moreover, missing data may affect the replicability of our results. Second, we did not have access to the primary SHIFT outcome measure (repetition of youth self‐harm, measured through hospital administrative data) due to privacy restrictions, which would have provided more objective information. Similarly, we relied on self‐report measures of parent criticism, and observational measures would provide more objective information regarding parent expressed emotion (Hooley & Parker, 2006). Third, most participating parents were mothers, and therefore, parent gender was not examined. Further research is needed to determine if the findings generalize to fathers or to criticism at the overall family level. Fourth, we used parent self‐report of criticism, whereas coded interviews or speech samples are the gold standard for assessing parent criticism and provide more objective information (Hooley & Parker, 2006). Fifth, the sample consisted primarily of participants who identified as White. Although the sample composition is similar to United Kingdom demographics, the results may not generalize to other ethnic groups, particularly given differences in cultural norms regarding expressed emotion (O'Driscoll, Sener, Angmark, & Shaikh, 2019). Sixth, we did not have information on levels of parent criticism prior to youth entry into the study, and we were therefore unable to examine earlier, preclinical trajectories of parent criticism. Seventh, we did not test whether change in parent criticism is a mediator of youth treatment outcomes. Finally, the present analyses were not specified in the original trial protocol and should therefore be considered exploratory and in need of replication.

## Conclusion

Parent criticism is a construct of long‐standing interest in youth psychopathology research and the present findings support continued investigations of new intervention approaches that may decrease criticism in clinic‐referred families seeking support for youth self‐harm. Assessment of parent criticism at baseline and throughout treatment may provide important context regarding parents' own mental distress and youth treatment prognosis (in the case of increasing parent criticism). At present, there is insufficient evidence to recommend a personalized decision regarding family therapy or usual outpatient care with regard to its potential to decrease parent criticism.

## Ethical considerations

SHIFT was approved by the UK NHS National Research Ethics Service. Youth and parents provided written informed consent. The present analysis was approved by the Research Ethics Board at the Centre for Addiction and Mental Health. Pseudo‐anonymized individual participant data was transferred securely subject to a formal Data Sharing Agreement.

**Key points**

- Parent criticism is associated with youth self‐harm and predicts a poor response to treatments for a range of psychiatric disorders.
- Most parents reported levels of criticism that remained elevated across youth treatment for self‐harm, though some showed sharply decreasing, sharply increasing, or stable low trajectories of criticism.
- Treatment type (family therapy or usual care) was not related to differences in the trajectories of parent criticism.
- Assessment of parent criticism at baseline and during treatment may identify parents whose own mental distress is increasing and whose youth may be less likely to respond to intervention for self‐harm.

## Supporting information


**Appendix S1.** Supporting information.

## Data Availability

Access to the data requires approval from the SHIFT study team and the University of Leeds.
