# Supplementary material for: Trajectories of parent criticism across treatment for youth self‐harm
Source: J Child Psychol Psychiatry. 2025 Mar 24;66(10):1449–60. doi: 10.1111/jcpp.14144 (PMC12447688; doi:10.1111/jcpp.14144)

**Supporting Information:**

***Trajectories of Parent Criticism across Treatment for Youth Self-Harm***

**Missing Data Handling**

**Additional Missing Data Comparisons.** Compared to participants with available data at the same time point, participants with missing criticism data at 3 months or 6 months were significantly more likely to be in TAU than in family therapy, χ^2^(1) = 9.36, *p* = .002, and χ^2^(1) = 18.15, *p* < .001 for those missing at 3 and 6 months, respectively; had significantly higher baseline parent mental distress, *t*(827) = 2.48, *p* = .013, and *t*(827) = 3.64, *p* < .001 for those missing at 3 and 6 months, respectively; endorsed less emotion relief reasons for self-harming at baseline, *t*(772) = -2.56, *p* = .011 for those missing at 3 months, and *t*(772) = -2.68, *p* = .007 for those missing at 6 months, respectively; and were more likely to endorse having communicated their suicide intent to someone at baseline for those missing criticism ratings at 3 months (but not 6 months), χ^2^(1) = 4.39, *p* = .036. Participants with and without missing parent criticism ratings at 3 and 6 months did not differ significantly in youth gender, age, or baseline suicidal ideation severity, depression, hopelessness, lethality of their self-harm, intent to die, or interpersonal influence reasons for self-harming (*p*s ≥ .05).

Those with missing outcome data at 12 months were significantly more likely to be in TAU than in family therapy, χ^2^(1) = 17.21, *p* < .001, were significantly older, *t*(830) = 2.50, *p* = .012, and had parents with significantly higher mental distress at baseline, *t*(827) = 2.63, *p* = .009. There were no significant differences between those with missing and complete outcome data at 12 months in terms of gender, having experienced physical or sexual abuse, use of medication at baseline, scores for suicidal ideation severity, depression, or hopelessness at baseline, or parent criticism ratings at any of the three time points (*p*s ≥ .05).

For outcome variables at 18 months, those with missing data were significantly more likely to be in TAU than in family therapy, χ^2^(1) = 9.96, *p* = .002, to be girls, χ^2^(1) = 4.63, *p* = .031, and to have experienced physical abuse by parents χ^2^(1) = 4.20, *p* = .041, or abuse that left marks, χ^2^(1) = 4.04, *p* = .044. Participants with missing data at 18 months also had significantly higher baseline parent criticism ratings, *t*(829) = 2.06, *p* = .040. There were no significant differences between those with missing and complete outcome data at 18 months in terms of age, having experienced sexual abuse, use of medication at baseline, scores for suicidal ideation severity, depression, hopelessness, or parent mental distress at baseline, or parent criticism ratings at 3 or 6 months (*p*s ≥ .05).

Below, we describe how missing data were handled for each of our three overarching hypothesis tests and provide a rationale for the choice of missing data handling.

**Hypothesis 1: Trajectories of Change in Parent Criticism.** Our pre-registered analysis plan included the use of full-information maximum likelihood (FIML) estimation to handle missing criticism ratings in the growth mixture modeling analysis. FIML is appropriate when data are missing completely at random or missing at random; however, there were indications that our data were not missing at random given that higher baseline criticism scores were associated with missing data on the same variables at 3 and 6 months. Our pre-registered analysis plan also included a sensitivity analysis using a pattern mixture model (Muthén & Asparouhov, 2011) in which intercepts and slopes for each class are regressed on a dummy coded dropout variable. This approach has been developed and recommended for situations in which missing data are non-ignorable, including in clinical trials (Muthén & Asparouhov, 2011). Following recommended practices (Muthén & Asparouhov, 2011), we re-ran our growth mixture model using a PMM and identified the best fitting number of classes using the Bayesian information criterion (BIC). We then compared the resulting model with our initial growth mixture model, which used full information maximum likelihood (FIML) estimation to handle missing data. The PMM analysis supported a 4-class model (see Table S2), and results were comparable to the original models under FIML; therefore, we retained the FIML growth mixture model for further analyses.

**Hypothesis 2: Treatment Comparisons by Class Membership.** This analysis used the 3-step method to compare latent classes on treatment condition (family therapy vs. treatment as usual) in which the latent class model is formed and class membership is regressed on treatment condition. All participants had available data on treatment condition, so no additional missing data handling was required (FIML was already used to estimate the latent classes with missing parent criticism values, and this approach was validated using a pattern mixture model, as described in the section for Hypothesis 1).

**Hypothesis 3: Comparing Classes on Baseline Variables.** Some participants were missing data on baseline variables but had available data on parent criticism at one or more time points. Our analyses for Hypothesis 3 used the R3STEP method in Mplus, which can aggregate results across multiply imputed data. We therefore imputed missing data for baseline auxiliary variables on which the classes were compared. The imputation was done in Mplus with 100,000 iterations, creating 20 imputed datasets. Twenty imputed datasets were selected because the fraction of missing information was low (<.001 - .224); therefore, additional imputations were unlikely to improve precision (Graham, Olchowski, & Gilreath, 2007). The following additional variables were used in the imputation, based on their association with missingness and/or with variables having missing data: treatment condition (family therapy vs. TAU), sex, and the following variables at 12 and 18 months: Beck Scale for Suicide Ideation, Children’s Depression Rating Scale, Hopelessness Scale, and parent General Health Questionnaire.

**Hypothesis 4: Comparing Classes on Treatment Outcomes.** We were unable to use imputed auxiliary variables at 12 and 18 months because the analysis of distal outcomes in Mplus is not able to aggregate results across multiply imputed datasets; therefore, we used full information maximum likelihood to handle missing data on variables at 12 and 18 months.

**Additional Details of Growth Mixture Model**

We encountered model convergence issues when using the Mplus default set-up for the two- through five-class models. We made the following model modifications to obtain convergence: For the two-class model, the correlation between intercept and slope was greater than 1, causing convergence issues. We fixed the correlation between the intercept and slope to .99 and the model converged. For the three-, four-, and five-class models, the variance of the slope was negative and non-significant. We therefore fixed the slope variance to zero and the models converged.

**Alternative Model Specifications and Shapes**

Our pre-registered analysis plan involved the use of growth mixture modeling (GMM) of parent criticism across treatment. We used the Mplus default of fixing residual variances across classes, rather than setting variances for intercepts and slopes to zero, which would have been too restrictive and not likely to represent the data. We also did not estimate the intercept and slope variances freely, as we did not have substantive rationale for which variances to free, and freeing all variances would have made the model extremely complex.

Following recommended practices,(Jung & Wickrama, 2008; van de Schoot, Sijbrandij, Winter, Depaoli, & Vermunt, 2017) we also tested a latent class growth analysis (LCGA) as an alternative model specification, in which the variance of the intercept and slope are fixed to zero. In addition, because our use of three time points in our GMM can only identify linear trajectories, we also tested a longitudinal latent class analysis,(Feldman, Masyn, & Conger, 2009) which allows for estimation of non-linear change.

Within each model specification type, we identified the number of classes that fit best. We then compared the best fitting models from each model specification type based on: 1) convergence properties; 2) fit statistics; 3) residuals; 4) parsimony; and 5) interpretability.(Feldman et al., 2009)

For criticism, all models converged and identified interpretable classes. An examination of the LLCA plots and BIC values (see Table S3) did not show evidence of non-linear change, and LLCA is also less parsimonious (requiring the estimation of more parameters) than GMM or LCGA,(Feldman et al., 2009) so we rejected the LLCA specification. There were relatively few large residuals for the GMM (3 residuals ≥ 2) and none in the LCGA; however, we retained the GMM as the preferred model specification because BIC values indicated that it fit the data better than the LCGA (see Table S3) and the more restrictive nature of LCGA means that these models are generally seen as a preliminary step, with GMM preferred when possible.(Jung & Wickrama, 2008)

As shown in Table S3, the growth mixture model fit better than the more restrictive latent class growth analysis model and the non-linear longitudinal latent class analysis model.

**Sensitivity Analyses**

**Removing outliers.** We re-ran the best-fitting growth mixture models excluding participants with extreme values on parent criticism (*z* ≥ |2.24|) in order to ensure that extreme values were not influencing the identification of classes. After excluding the 15 participants with extreme scores at one or more time points, we obtained a similar pattern of classes, with only one participant lost from each of the two smallest classes. Therefore, our smallest classes cannot be explained by participants having extreme values on parent criticism ratings. We therefore retained the results using the full sample.

**Deviations from Preregistered Analysis Plan**

**Threshold for Outliers.** In our pre-registration, we stated that we define outliers as ≥ 2.4 standard deviations, following recommended practices.(Aguinis, Gottfredson, & Joo, 2013) This is an error, and Aguinis and colleagues in fact recommend a threshold of *z* ≥ 2.24. We therefore used 2.24 as the threshold to define outliers for our sensitivity analysis, and re-ran the growth mixture model excluding participants who were outliers for criticism. This change was made prior to any exploration of outliers in our dataset.

**Omitting Some Control Variables in Baseline Comparisons of Criticism Classes.** In our pre-registration, we stated that we would include baseline psychotropic medication use among our control variables when comparing classes on baseline variables. In our initial analyses comparing criticism classes on baseline variables (following our pre-registered use of control variables), we observed extremely large estimates and standard errors for baseline psychotropic medication use, likely due to the small number of participants taking psychotropic medication at baseline, which led to small cell sizes for some analyses. Further investigation using saved class membership revealed that there were no participants in the Increasing or Decreasing classes who were taking psychotropic medication, which likely led to unreliable estimates. As a result, we omitted baseline psychotropic medication as a control variable in the comparison of baseline variables across classes. The pattern of results did not differ with and without baseline psychotropic medication included as a control variable. Similarly, after removing baseline psychotropic medication, we observed extremely large parameter estimates and standard errors for sex in the comparison of criticism classes on baseline parent mental distress and the SASII interpersonal influence scale. We removed sex as a control variable for these analyses and obtained a similar pattern of results.

**No Control Variables Used in Comparison of Classes on Treatment Outcome.** In our pre-registration, we stated that we would control for baseline values on the outcome of interest, as well as youth age, sex, history of abuse and psychotropic medication use, when comparing outcomes for youth and parent treatment response at 12 and 18 months. This was an oversight given that the appropriate 3-step method for distal outcomes in Mplus uses an equality test of means, rather than a regression of class membership on variables of interest. As a result, it is not possible to include control variables in our comparison of classes on the distal outcomes of treatment response, and all such analyses were conducted without the inclusion of these control variables. While it would have been possible to use saved class membership in a regression model, we favored the three-step method due to its ability to account for error in class membership given that entropy values were not high enough in our best fitting growth mixture model to allow the use of saved class membership.

**Use of Self-Harm as an Auxiliary Variable.** Our pre-registered analysis plan included the use of number of self-harm incidents at baseline, 12, and 18 months as auxiliary dependent variables on which to compare the classes. Upon obtaining the data, we determined that the number of self-harm incidents is categorical, not continuous (i.e., higher frequencies were collapsed into ranges, with 15 categories total). Because this number of categories exceeds the maximum number of categories permitted in Mplus, we omitted the number of self-harm incidents at baseline as an auxiliary variable on which to compare classes. At 12 and 18 months follow-up, we used a binary outcome (yes/no self-harm since the previous time interval, i.e., from baseline to 12 months, and from 12 to 18 months). It was not possible to use a binary self-harm variable to compare classes at baseline as all participants had engaged in self-harm.

**Growth Mixture Model of Parent Emotional Over-Involvement.** In addition to our primary analysis focused on parent criticism, our pre-registered plan included examining trajectories of parent emotional over-involvement (a second dimension of expressed emotion from the Family Questionnaire) using growth mixture modeling. After carrying out the growth mixture model for emotional over-involvement, the final model had low entropy values (< .60) suggested poor separation of classes for emotional over-involvement, which can lead to biased estimates in the analysis of auxiliary variables.(Asparouhov & Muthen, 2021) As criticism is the dimension of expressed emotion that has been most consistently related to differences in youth mental health outcomes (McCarty & Weisz, 2002) and the emotional over-involvement class separation was poor, we do not report the results of the growth mixture model, nor the comparisons of emotional over-involvement classes at baseline or follow-up.

References

Aguinis, H., Gottfredson, R. K., & Joo, H. (2013). Best-practice recommendations for defining, identifying, and handling outliers. *Organizational Research Methods*, *16*(2), 270–301.

Asparouhov, T., & Muthen, B. (2021). Auxiliary variables in mixture modeling: Using the BCH method in Mplus to estimate a distal outcome model and an arbitrary secondary model.

Feldman, B. J., Masyn, K., & Conger, R. D. (2009). New approaches to studying problem behaviors: A comparison of methods for modeling longitudinal, categorical adolescent drinking data. *Developmental Psychology*, *45*(3), 652–676.

Graham, J. W., Olchowski, A. E., & Gilreath, T. D. (2007). How many imputations are really needed? Some practical clarifications of multiple imputation theory. *Prevention Science*, *8*(206–213).

Jung, T., & Wickrama, K. A. (2008). An introduction to latent class growth analysis and growth mixture modeling. *Social and Personality Psychology Compass*, *2*(1), 302–317.

Kazdin, A. E., Rodgers, A., & Colbus, D. (1986). The Hopelessness Scale for Children: Psychometric characteristics and concurrent validity. *Journal of Consulting and Clinical Psychology*, *54*(2), 241–245.

McCarty, C. A., & Weisz, J. R. (2002). Correlates of expressed emotion in mothers of clinically referred youth: An examination of the five minute speech sample. *Journal of Child Psychology and Psychiatry*, *43*(6), 759–768.

Muthén, B., & Asparouhov, T. (2011). Growth modeling and non-ignorable dropout: Alternative analysis of the STAR*D antidepressant trial. *Psychological Methods*, *16*(1), 17–33.

van de Schoot, R., Sijbrandij, M., Winter, S. D., Depaoli, S., & Vermunt, J. K. (2017). The GRoLTS-checklist: Guidelines for reporting on latent trajectory studies. *Structural Equation Modeling*, *24*(3), 451–467.

Table S1

*Guidelines for Reporting on Latent Trajectory Studies (GRoLTS) Checklist*

| Item | Location in Manuscript |
| --- | --- |
| 1. Is the metric of time used in the statistical model reported? | p. 8 |
| 2. Is information presented about the mean and variance of time within a  wave? | p. 10 |
| 3a. Is the missing data mechanism reported? | p. 10 |
| 3b. Is a description provided of what variables are related to attrition/missing  data? | p. 10 & Supplement p. 1-2 |
| 3c. Is a description provided of how missing data in the analyses were dealt  with? | p. 9 &  Supplement p. 1-2 |
| 4. Is information about the distribution of the observed variables included? | p. 10 |
| 5. Is the software mentioned? | p. 8 |
| 6a. Are alternative specifications of within-class heterogeneity considered (e.g.,  LGCA vs. LGMM) and clearly documented? If not, was sufficient  justification provided as to eliminate certain specifications from  consideration? | Supplement p. 3-4 |
| 6b. Are alternative specifications of the between-class differences in variance–  covariance matrix structure considered and clearly documented? If not, was  sufficient justification provided as to eliminate certain specifications from  consideration? | Supplement p. 3 |
| 7. Are alternative shape/functional forms of the trajectories described? | Supplement p. 3-4 |
| 8. If covariates have been used, can analyses still be replicated? | p. 8 |
| 9. Is information reported about the number of random start values and final  iterations included? | p. 8 |
| 10. Are the model comparison (and selection) tools described from a statistical  perspective? | p. 8 |
| 11. Are the total number of fitted models reported, including a one-class  solution? | Table 2 |
| 12. Are the number of cases per class reported for each model (absolute sample  size, or proportion)? | Table 2 |
| 13. If classification of cases in a trajectory is the goal, is entropy reported? | Table 2 |
| 14a. Is a plot included with the estimated mean trajectories of the final solution? | Figure 1 |
| 14b. Are plots included with the estimated mean trajectories for each model? | Figure S1 |
| 14c. Is a plot included of the combination of estimated means of the final model  and the observed individual trajectories split out for each latent class? | Figure S2 |
| 15. Are characteristics of the final class solution numerically described (i.e.,  means, SD/SE, n, CI, etc.)? | Table S4 |
| 16. Are the syntax files available (either in the appendix, supplementary  materials, or from the authors)? | p. 8 |

Table S2

*Fit Indices for Growth Mixture Models Using Pattern Mixture Model for Data Not Missing at Random*

| Classes | BIC | BLRT *p* |
| --- | --- | --- |
| 1 | 10380.07 | - |
| 2 | 10360.50 | <.001 |
| 3 | 10361.54 | <.001 |
| 4 | **10357.12** | **<.001** |
| 5 | 10367.15 | .074 |

*Note.* Bold values indicate best model fit based on BIC. BIC = Bayesian information criterion; BLRT = bootstrap likelihood ratio test.

Table S3

*Comparison of Best-Fitting Models across Model Specifications Tested*

| Model Specification | Classes | BIC |
| --- | --- | --- |
| GMM | 4 | 10348.35 |
| LCGA | 4 | 10384.39 |
| LLCA | 2 | 10364.45 |

*Note.* GMM = growth mixture model; LCGA = latent growth curve analysis; LLCA = longitudinal latent class analysis.

Table S4

*Characteristics of Final Class Solutions for Growth Mixture Model of Criticism (n = 831)*

| Class | Parameter | *M* | *SD* | *p* | 95% CI |
| --- | --- | --- | --- | --- | --- |
| High with small decrease | I | 29.93 | 3.64 | <.001 | 29.17, 30.69 |
|  | S | -1.50 | 0.00 | <.001 | -2.00, -1.00 |
| Sharply decreasing | I | 29.90 | 3.64 | <.001 | 28.22, 31.59 |
|  | S | -7.14 | 0.00 | <.001 | -8.79, -5.50 |
| Low/stable | I | 19.26 | 3.64 | <.001 | 18.30, 20.23 |
|  | S | -0.33 | 0.00 | .153 | -0.78, 0.12 |
| Increasing | I | 22.55 | 3.64 | <.001 | 18.16, 26.94 |
|  | S | 5.09 | 0.00 | <.001 | 3.39, 6.79 |

*Note.* I = intercept; S = slope.

Table S5

*Multinomial Logistic Regressions of Criticism Class Membership on Control Variables (*n *= 831)*

|  | High with Small Decrease | | |  | Sharply Decreasing | | |  | Increasing | | |
| --- | --- | --- | --- | --- | --- | --- | --- | --- | --- | --- | --- |
| Variable | Estimate | *SE* | *p* |  | Estimate | *SE* | *p* |  | Estimate | *SE* | *p* |
| Age | -0.10 | 0.08 | .188 |  | 0.03 | 0.21 | .869 |  | -0.21 | 0.23 | .376 |
| Gender | -0.45 | 0.34 | .188 |  | 1.69 | 2.70 | .532 |  | -0.61 | 1.00 | .540 |
| Physical abuse by parent | **-1.44** | **0.32** | **<.001** |  | -0.13 | 0.88 | .880 |  | -0.92 | 0.93 | .324 |
| Physical abuse with marks | **-0.84** | **0.29** | **.004** |  | -0.33 | 0.70 | .643 |  | -1.16 | 0.82 | .153 |
| Sexual abuse | **0.56** | **0.17** | **.011** |  | 0.53 | 0.35 | .185 |  | 0.70 | 0.73 | .683 |

*Note.* Bold values indicate a significant difference from the reference class (Low/stable). Variables were entered in separate analyses.

**Figure S1**

*Plots of Estimated Mean Criticism Trajectories for Each Growth Mixture Model*

*
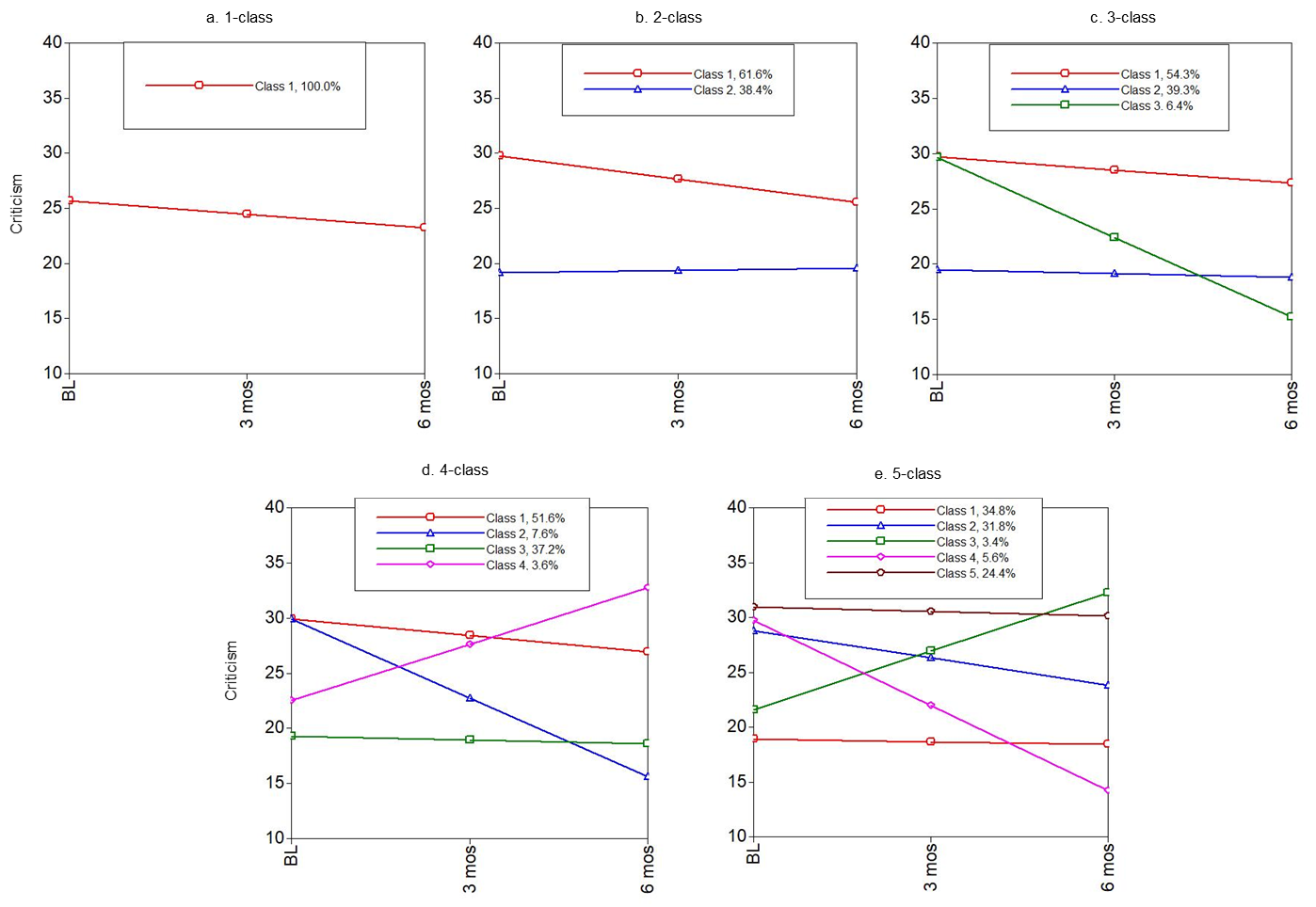
*

**Figure S2**

*Plots of Estimated Mean Criticism Trajectories and Individual Observed Scores by Class for Final 4-Class Model*


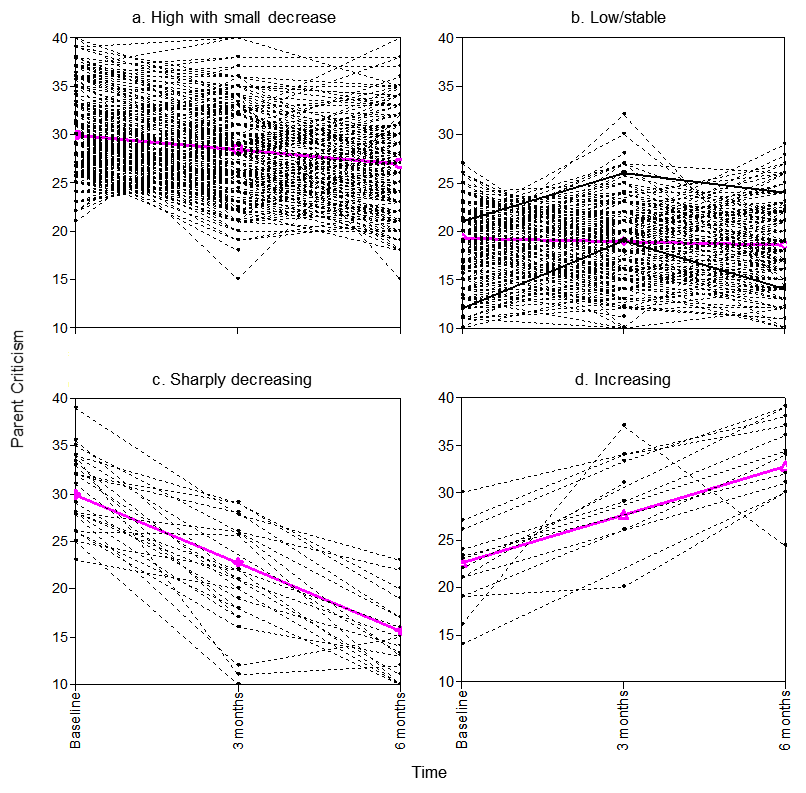

Supplement: Supplementary file 1 — Appendix S1. Supporting information. [file JCPP-66-1449-s001.docx]
